# Robust antibody and CD8^+^ T-cell responses induced by *P. falciparum* CSP adsorbed to cationic liposomal adjuvant CAF09 confer sterilizing immunity against experimental rodent malaria infection

**DOI:** 10.1038/s41541-017-0011-y

**Published:** 2017-04-10

**Authors:** Diego A. Espinosa, Dennis Christensen, Christian Muñoz, Sanjay Singh, Emily Locke, Peter Andersen, Fidel Zavala

**Affiliations:** 1 Department of Molecular Microbiology and Immunology, Johns Hopkins Malaria Research Institute, Johns Hopkins Bloomberg School of Public Health, Johns Hopkins University, Baltimore, MD USA; 2 Department of Infectious Disease Immunology, Statens Serum Institut, Copenhagen, Denmark; 3 Gennova Biopharmaceuticals Ltd., Pune, India; 4 PATH Malaria Vaccine Initiative, Washington, DC USA; 5 Present Address: Division of Infectious Diseases and Vaccinology, School of Public Health, University of California, Berkeley, Berkeley, CA USA

## Abstract

Despite several decades of extensive research, the development of a highly efficacious malaria vaccine has yet to be accomplished. While the RTS,S malaria vaccine candidate shows the potential to prevent a substantial number of clinical malaria cases, significant improvements in protective efficacy are still needed. Multiple studies have shown that RTS,S induces protective antibody and CD4^+^ T-cell responses, but limited or negligible CD8^+^ T cells. In this study, we evaluated the immunogenicity and protective capacity of full-length recombinant *Plasmodium falciparum* circumsporozoite protein administered with the novel cationic liposomal adjuvant system CAF09. Using newly developed transgenic rodent malaria parasites expressing the full-length *Plasmodium falciparum* circumsporozoite protein, we demonstrate that this liposome-based protein-in-adjuvant formulation is capable of inducing robust antibody and CD8^+^ T-cell responses that strongly inhibit parasite infection and development of liver stages, conferring durable sterilizing immunity. These findings underscore the potential of liposome-based adjuvants for inducing robust humoral and CD8^+^ T-cell responses and warrant further studies toward the development of novel subunit vaccine formulations with this adjuvant system.

## Malaria: A more effective vaccine

A vaccine consisting of parasitic proteins enveloped by fatty molecules provides comprehensive protection against malaria in a rodent model, Previous and current malaria vaccines concentrate on priming antibodies to recognize malarial infection, despite evidence that, by activating ‘killer’ CD8^+^ T cells, greater protection is conferred against the disease. Fidel Zavala, of the Johns Hopkins University, United States, and an international group of researchers developed their vaccine by encapsulating proteins from the malaria-causing parasite *Plasmodium falciparum* in fat-based carriers called liposomes. In past experiments, killer T cells recruited via this vaccine-type have effectively protected against other diseases. In this study, the vaccine induced both CD8^+^ T cell and antibody responses and provided significant immunity against *P. falciparum*-instigated malaria. As a highly efficacious vaccine against malaria is not yet available, this research will likely prove invaluable in guiding further studies.

## Introduction

CD8^+^ T cells are a major protective immune mechanism against intracellular infections caused by viruses, bacteria and parasites. While the protective capacity of these T cells is firmly established, the development of vaccines designed to induce CD8^+^ T-cell-mediated immunity has not advanced significantly, likely due to a lack of safe and efficient vaccine platforms capable of inducing robust and long-lived T-cell responses in humans.^1^ Attenuated viruses expressing foreign antigens are highly immunogenic and capable of inducing protective T-cell responses in animal models; however, they may not be as efficient in human vaccination. This is probably due to limitations imposed by pre-existing immunity, species specificity of recombinant viruses, and safety concerns that limit the magnitude of immunizing doses.^2–4^

All vaccines currently in use, as well as those in advanced development stages, are designed to induce protective antibody responses.^1^ And while humoral immunity is a highly efficient protective mechanism, it is reasoned that vaccines that are also capable of inducing CD8^+^ T cells will likely increase the protective efficacy of vaccine-induced antibody responses.^5, 6^ Indeed, CD8^+^ T cells will complement the humoral protective activity by exerting their anti-microbial effects against intracellular stages of infection, which are not commonly affected by circulating antibodies.

Malaria vaccines currently in preclinical and human clinical trials may greatly benefit from the development of new immunogenic constructs capable of inducing antibody and CD8^+^ T-cell responses. RTS,S, a subunit malaria vaccine candidate that is currently moving through the policy and regulatory process, is based on the *Plasmodium falciparum* circumsporozoite protein (*P. falciparum* CSP), incorporating the repeat and C-terminal regions of this antigen hybridized with the viral envelope protein of the hepatitis B virus (HBsAg). RTS,S was adjuvanted with AS01, consisting of monophosphoryl lipid A and QS21 incorporated into liposomes comprising dioleoylphosphatidylcholine and cholesterol.^7^ Phase 3 clinical trials demonstrated that among children 5 months to 17 months old, who received three doses of RTS,S/AS01, vaccine efficacy against clinical malaria ranged from about 51% over 12 months of follow-up from dose 1 to about 26% after approximately 4 years of follow-up. Vaccine efficacy was enhanced by a fourth dose given 18 months after dose 3. Among those who received a fourth vaccine dose, efficacy against clinical malaria was around 39% over roughly 4 years of follow-up.^8, 9^ The trials showed that RTS,S-induced immunity wanes over time, with significantly reduced efficacy 3 years post-immunization.^10^ Thus, while the results obtained with RTS,S are encouraging, it is generally accepted that improvements in the efficacy of this vaccine are still necessary.

Immunization with RTS,S induces robust anti-CSP antibodies and CD4^+^ T-cell responses that correlate with protection against sporozoite infection.^11–14^ Nevertheless, this vaccine does not appear to induce significant CD8^+^ T-cell responses among vaccinated individuals.^15–18^ This may be an important shortcoming of RTS,S as it has been extensively demonstrated in animal models that CD8^+^ T cells are highly efficient at eliminating parasite-infected hepatocytes.^19, 20^ To improve this aspect, prime-boost approaches using RTS,S and viral vectors or DNA constructs have been evaluated in clinical trials. However, these strategies did not result in considerable gains in overall protective immunity and vaccine efficacy in humans.^21–23^

The induction of CD8^+^ T cells by soluble protein-in-adjuvant systems has remained a difficult task, as poor results are obtained in different models.^1, 24^ However, a newly developed liposomal adjuvant (CAF09) has recently demonstrated remarkable capacity to induce strong antigen-specific antibody and CD8^+^ T cell responses.^25^ CAF09 is based on cationic surfactant dimethyldioctadecylammonium (DDA) incorporating immunostimulators monomycolyl glycerol (MMG) and polyinosinic:polycytidylic acid (poly I:C), which are ligands for Mincle and TLR-3 receptors, respectively. Importantly, antigen adsorbed to CAF09 liposomes efficiently induces cross-priming by dendritic cells (DCs), and the induction of antigen-specific CD8^+^ T cells has been documented with several different antigens on proteins or peptides.^25^ In addition, CD8^+^ T cells elicited by CAF09 in combination with the E7 antigen from HPV16 confer complete protection in a prophylactic skin tumor model and enhanced protection as measured by reduced tumor growth for all mice in a therapeutic tumor model.^25^

In this study, we characterized the protective capacity of immune responses induced by a full-length recombinant *P. falciparum* CSP (Pf rCSP) administered with CAF09. We demonstrate that immunizations with this protein-in-adjuvant formulation induce potent anti-CSP antibody responses as well as robust antigen-specific CD8^+^ T-cell responses. Using a newly developed transgenic *Plasmodium berghei* parasite strain expressing the entire *P. falciparum* CSP, we show that these immune responses are capable of strongly inhibiting sporozoite infection and confer sterile immunity in mice.

## Results

### Pf rCSP-CAF09 induces significant antibody and CD8^+^ T-cell responses

**Fig. 1:**
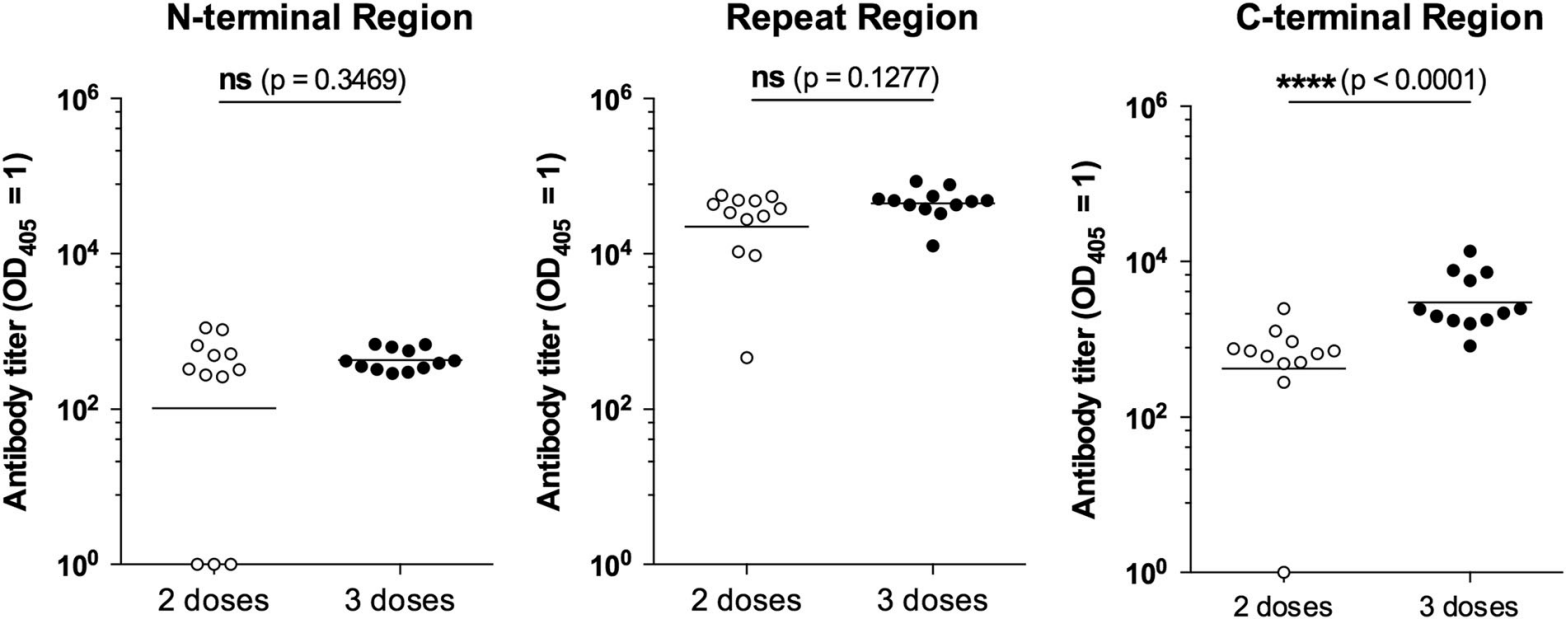
IgG titers against the N-terminal region, repeat region and C-terminal region of P. falciparum CSP. Mice immunized two or three times (*n* = 12 per group) with Pf rCSP-CAF09 developed comparable antibody titers against the *P. falciparum* CSP N-terminal peptide (**left**) and the repeat region peptide (**center**). However, three immunizations resulted in significantly higher titers against the C-terminal region (**right**). Titers are reported as the corresponding sera dilution at which OD_405_ was 1.0. Horizontal lines represent the geometric mean titer of the group. Abbreviations: ns, not significant; Pf rCSP, *P. falciparum* recombinant circumsporozoite protein; OD, optical density

Previous studies demonstrated that immunizations with cationic liposomes CAF09 induce strong humoral and T-cell immune responses.^25^ To determine the immunogenicity of Pf rCSP administered with CAF09 (Pf rCSP-CAF09), mice were immunized by intraperitoneal (i.p.) injection with this construct and immune responses evaluated after the 2nd and 3rd immunizations. In immunofluorescence assays (IFA), we found that mice immunized two or three times with Pf rCSP-CAF09 developed comparable antibody responses, capable of recognizing sporozoites from *P. falciparum* and *P. berghei* transgenic parasites expressing the entire *P. falciparum* CSP (Table 1 and Supplementary Fig. S1). The specificity of the antibody response was analyzed by enzyme-linked immunosorbent assays (ELISAs) using synthetic peptides representing the N-terminal region, repeat region, and C-terminal region of this protein. Antibody titers against the N-terminal region and the repeat region of the CSP were comparable in mice immunized two or three times (~250 and ~33,000 endpoint IgG titers, respectively) (Fig. 1a, 1b). However, mice immunized three times with Pf rCSP-CAF09 developed significantly higher antibody titers against the C-terminal region of *P. falciparum* CSP compared to mice that received only two immunizations (Fig. 1c). Importantly, these anti-CSP antibody responses were comparable or higher in magnitude than those obtained by immunization with Pf rCSP administered with other adjuvants, such as glucopyranosyl lipid adjuvant-stable emulsion, or with recombinant influenza (FluPf) and vaccinia (VacPf) viruses expressing selected regions of the *P. falciparum* CSP (Supplementary Fig. S2).

**Table 1:** Binding capacity of sera from Pf rCSP-CAF09-immunized mice to *P. falciparum* and P.b.-P.f. CSP-FL CD8CT transgenic sporozoites

Immunization	Qualitative score of tested sera—*P. falciparum* sporozoites					
	1:1000	1:4000	1:16000	1:64000	1:256000	1:1024000
Pf rCSP-CAF09 2×	+++	+++	++	++	+	−
Pf rCSP-CAF09 3×	+++	+++	+++	++	+	+/−
CAF09 3×	−	−	−	−	−	−

Immunization	Qualitative score of tested sera—P.b.-P.f. CSP-FL CD8CT sporozoites					
	1:1000	1:4000	1:16000	1:64000	1:256000	1:1024000
Pf rCSP-CAF09 2×	+++	+++	++	++	+	−
Pf rCSP-CAF09 3×	+++	+++	++	++	+	+/−
CAF09 3×	−	−	−	−	−	−

Pooled serum samples from mice immunized two or three times (*n* = 6/group) with Pf rCSP-CAF09 were incubated on slides coated with fixed sporozoites and stained with fluorescently labeled anti-mouse secondary antibody. Slides were qualitatively scored under a microscope

+++, strong;++, good;+, weak; and − no reactivity

Pf rCSP-CAF09, *P. falciparum* recombinant circumsporozoite protein in adjuvant CAF09; P.b.-P.f. CSP-FL CD8CT, *P. berghei–P. falciparum* CSP full-length CD8 epitope C-terminus transgenic parasite

**Fig. 2:**
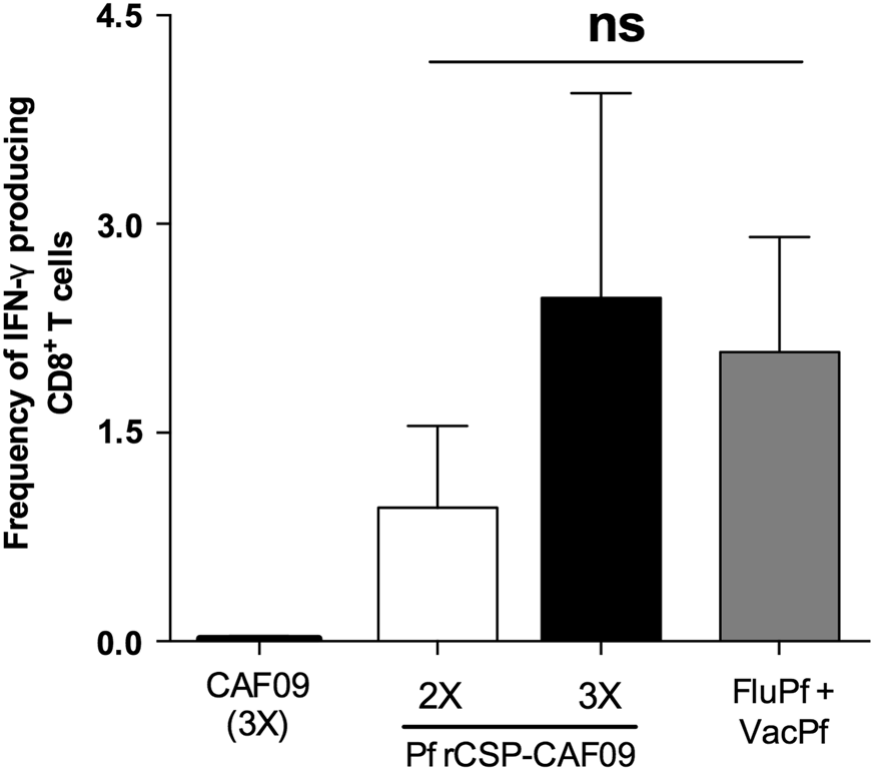
Antigen-specific CD8^+^ T-cell responses induced by Pf rCSP-CAF09 immunizations. CD8^+^ T-cell responses were assessed in spleens of mice 10 days after the last immunization with either two or three doses of Pf rCSP-CAF09 (*n* = 3 per group). Cellular responses were compared to those induced by a prime-boost regimen using recombinant influenza (FluPf) and vaccinia (VacPf) viruses expressing selected sequences of the *P. falciparum* CSP (*n* = 3). Mice immunized with CAF09 (3×) alone did not produce measurable T-cell responses. The production of IFN-γ by CD8^+^ T cells was assessed in ex vivo stimulation assays using LM1 cells pulsed with the synthetic peptide DYENDIEKKI. *Bars* represent means ± SEM. Abbreviations: *ns*, not significant; IFN-γ, interferon-gamma; SEM, standard error of the mean

The induction of CD8^+^ T-cell responses in spleen after immunization with Pf rCSP-CAF09 was evaluated in ex vivo assays using the synthetic peptide DYENDIEKKI, representing a cytotoxic epitope recognized by CD8^+^ T cells of C3H mice bearing the H-2K^k^ class I MHC.^26^ Notably, this epitope overlaps with the epitope recognized by human CD8^+^ T cells bearing HLA-B35.^27^ We determined the magnitude of the CD8^+^ T-cell responses against this epitope by assessing the production of epitope-specific IFN-γ by CD8^+^ T cells after incubation of 6 h with target cells coated with the synthetic peptide. We found that immunizations with two or three doses of Pf rCSP-CAF09 were capable of inducing similar CD8^+^ T-cell responses. More importantly, Pf rCSP-CAF09 elicited responses that were comparable in magnitude to those induced by the FluPf and VacPf prime-boost regimen, known to induce the most potent CD8^+^ T-cell responses (Fig. 2).^28, 29^

### Immunizations with Pf rCSP-CAF09 induce lasting sterilizing immunity that strongly inhibit infection of transgenic sporozoites expressing the *P. falciparum* CSP

Given the robust antibody and CD8^+^ T-cell responses induced by Pf rCSP-CAF09, we sought to determine whether these responses could inhibit sporozoite infection and development of liver stages. For this purpose, we developed a transgenic *P. berghei* parasite strain expressing the *P. falciparum* CSP protein. This transgenic *P. berghei* line expresses the full-length *P. falciparum* 3D7 CSP. However, in the region containing the sequence _359_DYANDIEKKI_368_, the A residue in position 361 was replaced by E to encode _359_DYENDIEKKI_368_, which is a murine H-2K^k^ cytotoxic epitope present in different *P. falciparum* strains such as 7G8 or T4. The sequence found in the 3D7 strain (_359_DYANDIEKKI_368_) is not recognized as a murine Class I MHC epitope.^26, 29^ This new transgenic parasite strain, *P. berghei-P. falciparum* CSP-full-length CD8 C-terminus (P.b.-P.f. CSP-FL CD8CT), develops normally in *Anopheles stephensi* mosquitoes and can efficiently infect naïve mice through mosquito bites (Supplementary Table S1). P.b.-P.f. CSP-FL CD8CT parasites can be specifically targeted by CD8^+^ T cells raised against the cytotoxic epitope _359_DYENDIEKKI_368_ using recombinant PfFlu and PfVac viruses. Critically, cytotoxic responses induced with these viruses were unable to inhibit liver stages of a similar transgenic *P. berghei* strain expressing the wild-type (WT) *P. falciparum* 3D7 CSP (i.e. with the unmodified _359_DYANDIEKKI_368_ epitope) (Supplementary Fig. S3). Parasites expressing the wild-type or the mutated 359-368 CSP 3D7 epitope were generated following the same methodological approach shown in Supplementary Fig. S4, and displayed comparable infectivity.

**Fig. 3:**
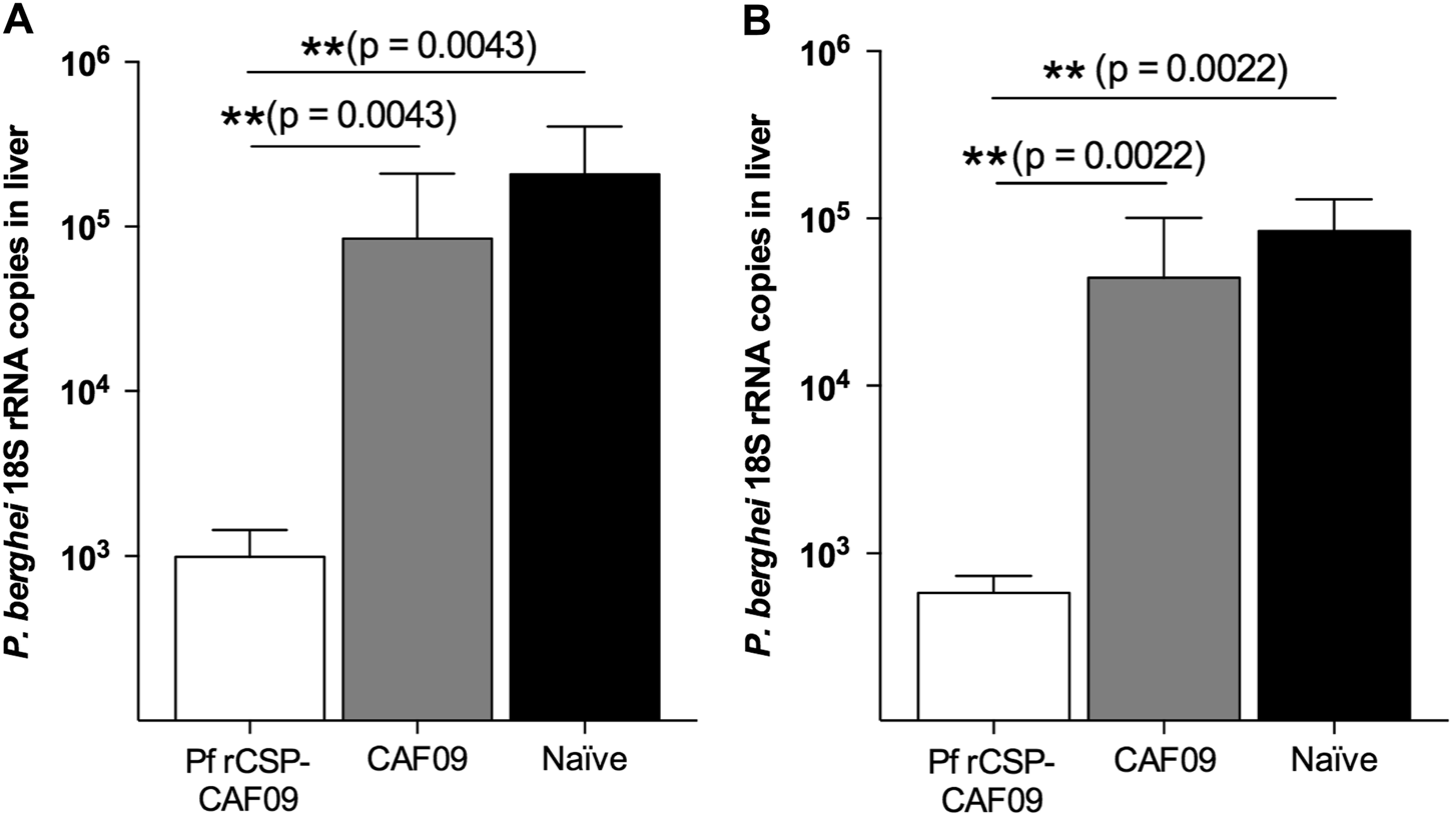
P.b.-P.f. CSP-FL CD8CT parasite liver burdens in mice immunized with Pf rCSP-CAF09. Mice immunized **a** two times or **b** three times with Pf rCSP-CAF09 significantly inhibited sporozoite infection compared to adjuvant-only (CAF09) or naïve controls. Geometric mean with 95% confidence interval; *n* = 6 per group. Abbreviations: Pf *rCSP-CAF09*, *P. falciparum* recombinant circumsporozoite protein in adjuvant CAF09; P.b.-P.f. CSP-FL CD8CT, *P. berghei–P. falciparum* CSP full-length CD8 epitope C-terminus transgenic parasite; rRNA, ribosomal RNA; *RT-qPCR* reverse-transcription quantitative real-time polymerase chain reaction

**Fig. 4:**
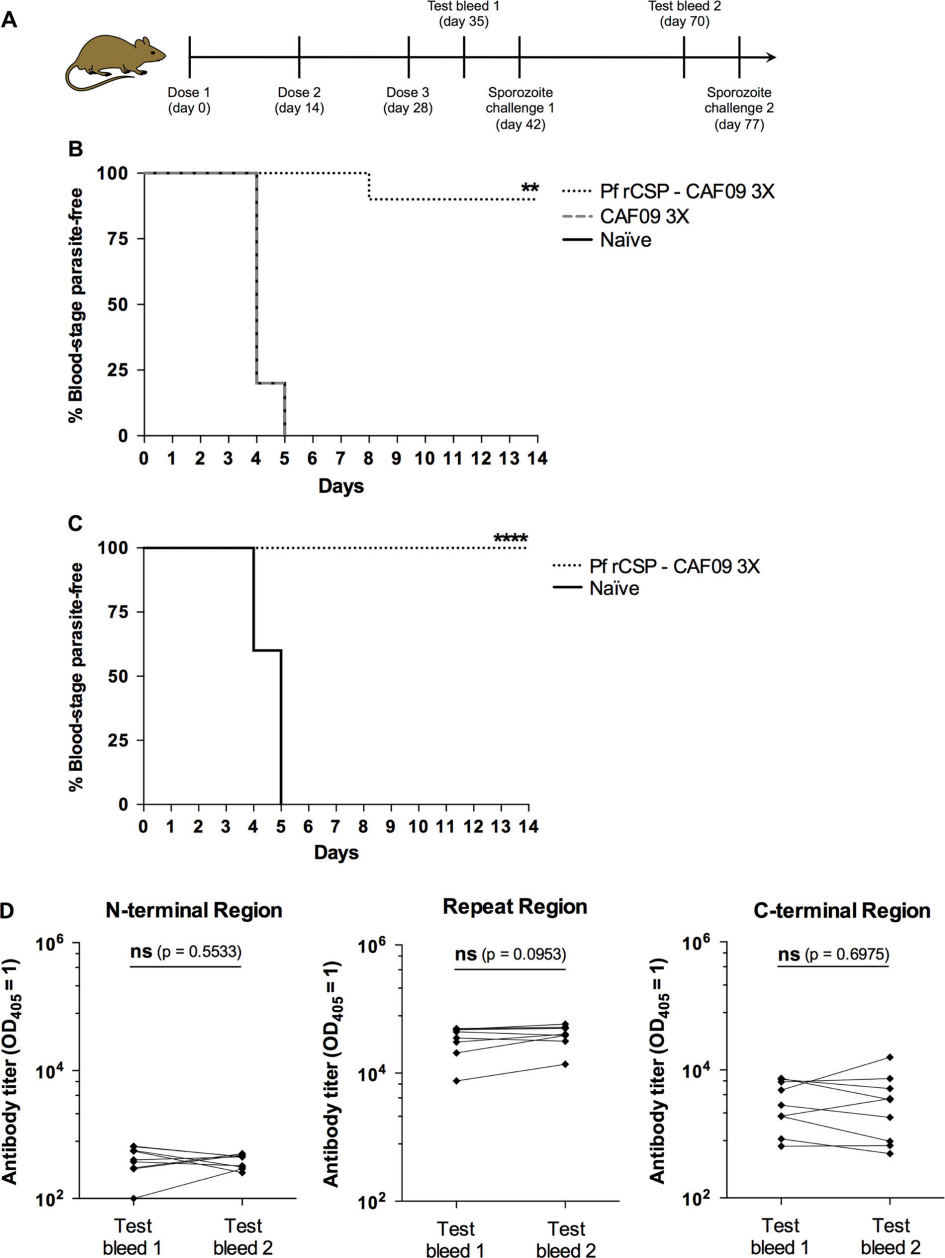
Assessment of sterile protection in Pf rCSP-CAF09-immunized mice against sporozoite infection. **a** Mice immunized three times with Pf rCSP-CAF09 were challenged with P.b.-P.f. CSP-FL CD8CT chimeric sporozoites delivered by five infectious mosquito bites. Four days after challenge, daily blood smears were taken and analyzed under a microscope until day 14. **b** Nine out of ten mice (90%) developed sterile immunity when challenged 2 weeks after the last immunization (*n* = 10). **c** Five weeks later, the nine mice that were sterilely protected in **b** were challenged again, with none of the animals developing blood-stage parasitemia. Kaplan–Meier plots show the time to detection of parasites in the blood for each group after the challenge (*n* = 10 for Naïve group). Data were analyzed for statistical significance using Log-rank (Mantel-Cox) test **P < 0.01; ****P < 0.0001. **d** Antibody titers against different regions of CSP were evaluated in mice immunized three times with Pf rCSP-CAF09 before and after sporozoite challenge (Test bleed 1 and Test bleed 2, respectively). IgG levels against the N-terminal region, repeat region and C-terminal region of CSP did not change significantly in sterilely protected mice during the 5-week interval. Titers are reported as the corresponding sera dilution at which OD405 was 1.0 and compared for significance using a paired Student’s *t*-test; *n* = 9. Abbreviations: *ns*, not significant; Pf rCSP-CAF09, *P. falciparum* recombinant circumsporozoite protein in adjuvant CAF09; OD, optical density

Previous research had indicated that recombinant PfFlu and PfVac viruses could elicit potentially protective immune CD8^+^ responses in mice.^29^ Thus, based on these studies, as well as on the fact that only P.b.-P.f. CSP-FL CD8CT liver stages can be specifically targeted by CD8^+^ T cells raised against the cytotoxic epitope _359_DYENDIEKKI_368_, this transgenic line was used to evaluate the protective capacity of Pf rCSP-CAF09. Mice were immunized with two doses (Fig. 3a) or three doses (Fig. 3b) of Pf rCSP-CAF09 and then challenged by i.v. injection with P.b.-P.f. CSP-FL CD8CT sporozoites. Forty-two hours after parasite infection, the liver of control and experimental mice were excised to measure parasite burden by reverse transcription followed by quantitative real-time PCR (RT-qPCR). In both experimental groups, the results showed a large reduction in liver parasite burden (~2 log_10_), compared to mice receiving adjuvant only or naïve controls. To establish whether Pf rCSP-CAF09 could confer sterile immunity, we challenged immunized mice with sporozoites delivered by infectious mosquito bites and monitored the development of blood-stage parasites (Fig. 4a). This challenge route is more physiologically relevant than i.v. sporozoite injection and arguably a better model for testing vaccine efficacy. Mice immunized three times with Pf rCSP-CAF09 were challenged against five infectious mosquito bites for 10 min. Four days later, daily blood smears were taken to determine the presence of blood-stage parasites until day 14. Notably, we found that 90% (9 out of 10) of Pf rCSP-CAF09 immunized mice did not develop blood-stage parasitemia during the 14-day follow-up period. All mice receiving only adjuvant, as well as the naïve controls, became positive for blood-stage infection by day 5 after challenge (Fig. 4b).

To determine if the observed sterile protection could persist over time, the nine mice that did not develop blood-stage parasitemia upon infectious mosquito bite were challenged again 5 weeks later (7 weeks after the last immunization). Remarkably, none of the Pf rCSP-CAF09-immunized mice developed blood-stage infection, while parasitemia in all of the age-matched naïve controls became patent by day 5 after challenge (Fig. 4c). Serum samples from mice that developed sterile immunity were analyzed regarding their epitope specificity at 1 and 6 weeks after the 3rd immunization of Pf rCSP-CAF09. Regardless of the CSP region under evaluation, we did not observe significant differences in antibody titers between 1 and 6 weeks post-immunization (Fig. 4d). However, we observed a slight increase in overall antibody levels against the CSP repeat region, with geometric mean titers of ~330,000 and ~370,000 at 1 and 6 weeks after immunization, respectively.

### CD8^+^ T cells play a significant role in reducing the liver parasite burden in Pf rCSP-CAF09-immunized mice

**Fig. 5:**
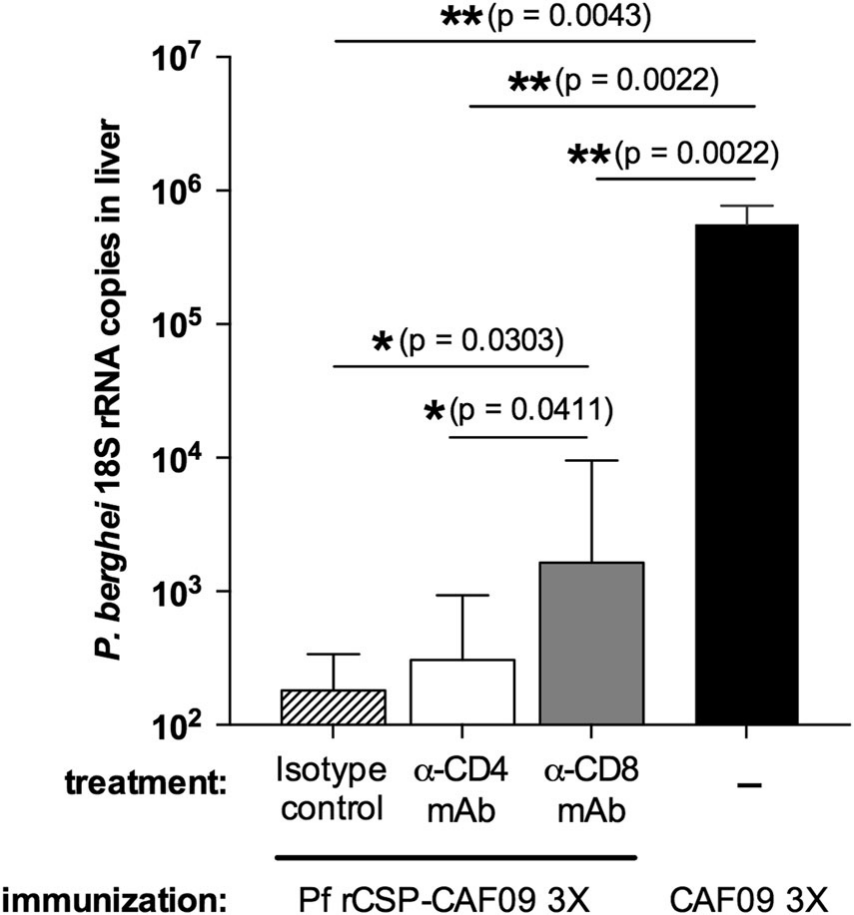
P.b.-P.f. CD8CT transgenic sporozoite liver infection in T-cell depleted Pf rCSP-CAF09-immunized mice. Mice immunized three times with Pf rCSP-CAF09 had a dramatically lower parasite liver burden than those vaccinated with adjuvant only (CAF09 3×). The depletion of CD8^+^ T cells significantly impaired their capacity to inhibit parasite liver stages compared to mice treated with an isotype control antibody or with a CD4^+^ T-cell depleting antibody. Geometric mean with 95% confidence interval; *n* = 6/group. Abbreviations: Pf rCSP-CAF09 *P. falciparum* recombinant circumsporozoite protein in adjuvant CAF09; P.b.-P.f. CSP-FL CD8CT, *P. berghei–P. falciparum* CSP full-length CD8 epitope C-terminus transgenic parasite; rRNA, ribosomal RNA; RT-qPCR reverse-transcription quantitative real-time polymerase chain reaction; α-CD4 mAb, anti-CD4 monoclonal antibody; *α-CD8 mAb* anti-CD8 monoclonal antibody

To evaluate the possible direct anti-parasite activity that vaccine-induced T cells may have in the presence of high antibody titers, mice immunized with Pf rCSP-CAF09 were treated with anti-CD4 or anti-CD8 antibodies to deplete the respective T-cell subsets. After the third immunizing dose, and 4 days before i.v. sporozoite challenge, some mice were treated twice with 150 μg of anti-CD4 or anti-CD8 monoclonal antibodies (Supplementary Fig. S5). Immunized mice treated with isotype control antibody had a marked reduction in liver parasite burden (~3 log_10_) compared to the adjuvant-only control (Fig. 5). Treatment with anti-CD4 antibodies did not reduce this protection, whereas anti-CD8 antibody-treated animals had a significantly higher liver parasite burden compared to mice treated with anti-CD4 antibody or the isotype control indicating that besides antibodies, immunization-induced CD8^+^ T cells play a major protective role in immunity induced by Pf rCSP-CAF09 (Fig. 5).

## Discussion

The development of a fully-efficacious protective vaccine is a top priority in the fight against malaria. In the RTS,S phase 3 trial, the vaccine candidate demonstrated a reproducible -yet partially- protective effect, ranging from 51% after 12 months follow-up from dose 1 to 39% after a fourth dose and approximately 48 months follow-up.^10^ This indicates that a virus-like particle (VLP)-in-adjuvant formulation can induce some level of protective humoral immune responses capable of preventing infection of hepatocytes. However, improvements in vaccine efficacy are still needed, and this may be accomplished by developing vaccines capable of inducing wide-breadth antibody responses and CD8^+^ T cells that can exert their protective effect against intracellular liver stages of *P. falciparum* infection. In this study, we demonstrate that immunizations with a single construct, consisting of full-length Pf rCSP in combination with the cationic liposomal adjuvant system CAF09, induce robust humoral and CD8^+^ T-cell responses that are capable of conferring durable sterilizing immunity against sporozoite infection.

Mice immunized with Pf rCSP-CAF09 developed high antibody titers, capable of binding different regions of the *P. falciparum* CSP as well as *P. falciparum* sporozoites and transgenic *P. berghei* sporozoites expressing the *P. falciparum* CSP. Interestingly, mice immunized two or three times with Pf rCSP-CAF09 developed comparable IgG titers against the *P. falciparum* CSP repeat region. However, three immunizations resulted in higher IgG titers against the N-terminal and C-terminal regions of the *P. falciparum* CSP. And while the potentially protective effect of antibodies against the C-terminal region of CSP is yet to be determined, we recently demonstrated that antibodies against the N-terminal region of this protein can significantly inhibit sporozoite infection.^30^ The fact that antibody titers persisted for several weeks after the last immunization of a 3-dose regimen is indicative of the enduring humoral responses elicited by Pf rCSP-CAF09. In this regard, findings from the recently completed RTS,S phase 3 clinical trial showed that anti-CSP antibody titers are indicative of the strength and duration of RTS,S/AS01-induced protective efficacy,^31^ thus underscoring the importance of robust and long-lived humoral responses. Additional studies should be useful to assess the kinetics of Pf rCSP-CAF09-induced anti-CSP antibodies over an extended follow-up period and to establish if comparable responses can be evoked by similar protein-in-adjuvant formulations. Nonetheless, our studies indicate that the antibody titers obtained with Pf rCSP-CAF09 are entirely comparable or better than those obtained with the same Pf rCSP antigen and immunization dose (20 μg) in combination with different adjuvant systems.^32^ Most importantly, only immunizations with CAF09 were capable of inducing strong CD8^+^ T-cell responses.

Indeed, our results indicate that, in addition to eliciting strong antibody responses, immunizations with two or three doses of Pf rCSP-CAF09 induce strong antigen-specific CD8^+^ T cells. Remarkably, these responses are comparable to those elicited by a prime-boost immunization using recombinant influenza virus for priming followed by recombinant vaccinia virus as booster, a protocol optimized to induce high-frequency antigen-specific CD8^+^ T cells against epitopes present in influenza virus, *P. berghei* and *P. falciparum* CSP.^28, 29^ Differently, studies in our laboratory with recombinant CSPs together with other adjuvant systems have consistently shown that these immunizations induce poor or no CD8^+^ T-cell responses. In this regard, it is also worth mentioning that the RTS,S vaccine does not elicit CD8^+^ T cells among vaccinated individuals, thus underscoring the limited ability of protein-in-adjuvant formulations to induce cytotoxic responses.^11^

For this study, we developed a new transgenic *P. berghei* strain expressing the full-length *P. falciparum* 3D7 CSP, incorporating the H-2K^k^ cytotoxic epitope DYENDIEKKI, naturally found in the C-terminal region of the CSP of different *P. falciparum* strains, such as 7G8 and T4, but not 3D7. The P.b.-P.f. CSP-FL CD8CT transgenic parasites develop normally in both mice and mosquitoes, and produce highly infectious sporozoites. Naïve mice exposed to the bites of three P.b.-P.f. CSP-FL CD8CT-infected mosquitoes develop blood-stage parasitemia within 5 days after exposure. Critically, CD8^+^ T cells induced by FluPf and VacPf viruses inhibited the development of liver stages of P.b.-P.f. CSP-FL CD8CT parasites expressing the cytotoxic epitope DYENDIEKKI but had no effect on parasites that expressed the *P. falciparum* CSP containing the sequence DYANDIEKKI. These data indicated that CD8^+^ T cells are protective only against the parasite expressing the CSP with the cytotoxic pertinent epitope, providing the rationale for using P.b.-P.f. CSP-FL CD8CT sporozoites for the assessment of Pf rCSP-CAF09-induced protective immune responses. The low level of antibodies induced after immunization with recombinant viruses, which are also known to be protective, likely explains the absence of protection in virus-vaccinated mice challenged with the parasite strain that does not express the DYENDIEKKI epitope.

Mice immunized with either two or three doses of Pf rCSP-CAF09 were able to significantly inhibit P.b.-P.f. CSP-FL CD8CT sporozoite infection and/or liver stage development with comparable efficiency, underscoring the potency of the immune response elicited by this protein-in-adjuvant formulation. To establish if Pf rCSP-CAF09 could confer sterilizing immunity against sporozoite infection, mice were challenged by infectious mosquito bites. In the first of these experiments, 90% of immunized mice did not develop blood-stage parasitemia. Most important, all sterilely protected mice were fully protected against an additional mosquito bite challenge 5 weeks later. This robust protection cannot be attributed to a boosting effect by sporozoites during the first experiment because the number of parasites delivered by mosquito bites, which is estimated to be just a few dozen,^33^ is considerably lower than the amounts required to boost CD8^+^ T-cell responses and to overcome T-cell self-regulation. As described in our previous studies, tens of thousands sporozoites are needed to boost established CD8^+^ T-cell responses and this booster dose needs to be administered at least 4 weeks after the last immunization.^34^ Furthermore, the fact that antibody titers against the different regions of CSP did not increase after sporozoite challenge, as shown in Fig. 5, also indicates that antibody responses are not boosted after sporozoite challenge. Thus, given the stringency of this challenge model, these findings emphasize the outstanding protective capacity of Pf rCSP-CAF09-induced immune responses. Notably, similar studies assessing the protective capacity of full-length *P. falciparum* CSP immunizations in combination with different adjuvants did not achieve the same degree of protection and enduring immunity as Pf rCSP-CAF09.^32, 35^

Given the importance of CD8^+^ T cells against pre-erythrocytic stages of malaria, we sought to determine the relative contribution of cytotoxic CD8^+^ T cells induced by Pf rCSP-CAF09 immunizations against parasite liver stages. Treatment of Pf rCSP-CAF09-immunized mice with anti-CD8 antibody significantly decreased the vaccine-induced protective effect. In contrast, the depletion experiments suggest that CD4^+^ T-cell responses are not critical effectors, as treatment with anti–CD4 did not affect the overall anti-parasite effect in immunized mice. The fact that in mice treated with anti-CD8 antibody, the parasite burden is 1 log_10_ higher than in CD4^+^ T-cell depleted and control mice indicates that Pf rCSP-CAF09-induced CD8^+^ T cells have a major anti-parasite effect. However, it is worth mentioning that the magnitude of the antibody responses induced by Pf rCSP-CAF09 may still understate the strength of the role of T-cell responses induced by these immunizations.

The vaccination strategy used in the present study involves i.p. immunizations, which for a human malaria vaccine may not be the preferred administration route. However, previous research indicated that this route of immunization is superior than subcutaneous (s.c.) or intramuscular (i.m.) routes for the induction of CD8^+^ T-cell responses.^25, 36^ This has to do with the ability of CAF09-adjuvanted vaccines to rapidly drain to the lymph nodes and spleen, which is required for cross-priming of CD8α^+^ DCs residing in the lymphoid tissues.^36^ Nevertheless, novel liposome systems incorporating poly I:C are currently under development and aimed to facilitate lymph node targeting after s.c./i.m. immunizations.

In conclusion, the most prominent finding of this study is that Pf rCSP-CAF09 was capable of inducing both strong antibody and CD8^+^ T-cell responses. In general, this is not observed upon immunization with subunit vaccines based on recombinant proteins, which are known to mainly induce antibodies or CD4^+^ T-cell responses, or with recombinant viruses, which preferentially induce T-cell responses.^1^ These findings highlight the potential of liposome-based approaches for inducing robust humoral and CD8^+^ T-cell responses, and warrant further research for vaccine development.

## Materials and methods

### Mice

Five-week old to 8-week old female C3H/HeNCr MTV and C57BL/6 mice were purchased from Charles River (Frederick, MD). Animals were tagged and distributed randomly in different cages following procedures recommended by the Institutional Animal Care and Use Committee (IACUC) of The Johns Hopkins University. The size of experimental and control groups to allow proper statistical analysis was based on estimates of anticipated variation of rodent models of malaria infection and on prior experience with this experimental system. The investigators were not blinded to analysis. All experimental procedures involving animals were approved by the IACUC of The Johns Hopkins University.

### Recombinant *P. falciparum* CSP and CAF09

The full-length Pf rCSP used for all immunizations was manufactured by Gennova Biopharmaceuticals (Pune, India) and kindly provided for this study by the PATH Malaria Vaccine Initiative. This protein is based on *P. falciparum* CSP from the Indian strain IND637HDD1 (GenBank: AAN87606.1) and it contains the murine H-2K^k^ cytotoxic epitope _373_DYENDIEKKI_383_, which is also present in other *P. falciparum* reference strains, including 7G8 and T4. These strains express the same epitopes in the repeat domain and the amino acid sequence of this recombinant protein is over 90% identical to the CSP sequences of the *P. falciparum* reference strains 3D7 and 7G8. Its use in pre-clinical studies has been previously reported.^32^ The cationic adjuvant formulation CAF09 was developed by the Statens Serum Institut (Copenhagen, Denmark) and it consists of DDA-liposomes stabilized with MMGs-1 in combination with Poly(I:C). The use of this formulation for animal immunizations has been previously reported.^25^

### Immunizations

C3H/HeNCr MTV mice (H-2K^k^) were immunized with Pf rCSP in combination with CAF09. For each immunization, 20 µg of Pf rCSP were mixed with CAF09 (DDA, 250 µg/dose, MMG-1, 50 µg/dose and Poly(I:C), 0.5 mg/ml) and administered by i.p. injection on study days 0 and 14 (2-dose regimen) or on days 0, 14 and 28 (3-dose regimen). The injection volume of each immunization was 0.2 ml.

### Recombinant viruses

Recombinant influenza and vaccinia viruses expressing selected sequences of the *P. falciparum* CSP were used to induce CD8^+^ T-cell responses in C3H/HeNCr MTV mice. Animals were immunized with 5 × 10^3^ viral particles of recombinant influenza (FluPf) expressing the peptide KPKDELDYENDIEKKICKMEKCS encoding a region of the *P. falciparum* CSP C-terminus and boosted with 5 × 10^6^ plaque forming units of recombinant vaccinia (VacPf) expressing the full-length *P. falciparum* 7G8 CSP gene. Immunizations were administered by i.p. injection on study days 0 and 14.

### Assessment of anti-CSP antibody titers

To assess antibody responses-induced Pf rCSP-CAF09 immunizations, polyclonal sera from immunized mice were evaluated 10 days after the last dose of each immunization regimen. We used an ELISA against synthetic peptides representing the N-terminal region (YGSSSNTRVLNELNYDNAGTNLYNELEMNYYGKQENWYSLKKNSRSLGENDDGNNEDNEKLRKPKHKKLKQPADHHHHHH), the repeat region [NANP]_7_ or the C-terminal region (SDKHIKEYLNKIQNSLSTEWSPCSVTCGNGIQVRIKPGSANKPKDELDYENDIEKKICKMEKHHHHHH) of *P. falciparum* CSP. Briefly, MaxiSorp^®^ ELISA plates (Thermo Scientific Nunc, Rochester, NY) were coated with 100 µl of synthetic peptide (1 µg/ml) and incubated overnight at room temperature. Plates were then washed and incubated with a 1% (w/v) bovine serum albumin (BSA) (Sigma-Aldrich, Saint Louis, MO)/1× phosphate buffered saline (PBS) solution (1% BSA-PBS) for an hour at room temperature. After another washing step, wells were incubated for 1 h at room temperature with serial dilutions of polyclonal sera. Plates were then washed and incubated for an hour at room temperature with a peroxidase-labeled goat anti-mouse (IgG H+L) secondary antibody (KPL, Gaithersburg, MD) at 0.5 µg/ml in 1% BSA-PBS. The assay was developed using a 2,2′-Azino-di(3-ethylbenzthiazoline-6-sulfonate) horseradish peroxidase substrate kit (KPL, Gaithersburg, MD), according to the manufacturer’s specifications. Antibody titers are reported as the corresponding sera dilution at which the optical density at 405 nm (OD_405_) was 1.0. Values were determined using a nonlinear regression with an asymmetric sigmoidal curve (GraphPad Prism 6 software).

### Immunofluorescence assays

Indirect IFA were used to assess anti-CSP titers in *P. falciparum* or transgenic sporozoites. In brief, poly-L-lysine-treated slides (Tekdon Inc., Myakka City, FL) were coated with a sporozoite suspension (4–6 × 10^5^ parasites/ml) and allowed to air dry at room temperature. Polyclonal serum samples were then diluted in 1% BSA-PBS and incubated on slides for 30 min at room temperature in a humidity chamber. Slides were then washed with PBS-1% BSA and a secondary-antibody solution [AlexaFluor 488 F(ab′)_2_ fragment of goat anti-mouse IgG(H+L); 2 mg/ml; Invitrogen] was added for 30 min at room temperature. Fluorescent sporozoites were visualized using an upright fluorescence microscope (Nikon Eclipse 90i).

### Evaluation of CD8^+^ T-cell responses

CD8^+^ T-cell responses in mouse spleens were assessed 2–3 weeks after the last immunization using an ex vivo stimulation assay. Single-cell suspensions (2 × 10^6^ cells/well) were incubated with LM1 (H-2K^k^) cells (5 × 10^5^ cells/well),^37^ pulsed with or without the synthetic peptide DYENDIEKKI (8 µM). Incubations were performed for 5–6 h at 37 °C in the presence of Brefeldin A and Monensin (BD Biosciences, San Diego, CA). Cells were surface stained with APC-anti-CD8 (eBioscience, San Diego, CA) and then treated with Cytofix/Cytoperm Fixation/Permeabilization Kit (BD Biosciences). FITC-anti-IFN-γ (eBioscience, San Diego, CA) was then used for intracellular staining.

### Cell depletion

To evaluate the anti-parasite effect of Pf rCSP-CAF09-induced T cells upon sporozoite infection, CD4^+^ T cells or CD8^+^ T cells were depleted by i.p. injections with 150 μg of anti-CD4 antibody (clone GK1.5) or anti-CD8 antibody (clone YTS 169.4), respectively. Antibody injections were administered on two consecutive days.

### Transgenic parasites

We developed a transgenic *P. berghei* strain in which the (WT csp gene was replaced with a *P. falciparum* CSP construct. A 784-bp restriction fragment encompassing base pairs 246 to 1029 of the *P. berghei-P. falciparum* CSP N-terminus csp chimeric gene was excised from plasmid pIC-CSPPfNT^30^ using restriction enzymes BbsI and PacI (New England Biolabs, Ipswich, MA). This portion was then replaced with a 943-bp fragment, which was released using the same restriction enzymes from the plasmid pHZ-PfCSP. Thus, the csp gene (1188 bp) in the resulting plasmid, pIC-CSPfFL-CD8CT, consists of a full-length *P. falciparum* 3D7 CSP in which the signal sequence has been replaced with the one of the *P. berghei* CSP (base pairs 1 to 69). In addition, a single nucleotide in the csp gene of plasmid pIC-CSPfFL-CD8CT was replaced to incorporate a cytotoxic epitope that is not present in the *P. falciparum* 3D7 strain. This change was introduced in position 1079 by site-directed mutagenesis using a QuikChange II XL Site-Directed Mutagenesis Kit (Agilent Technologies, La Jolla, CA). We then excised the csp gene from pIC-CSPfFL-CD8CT as a KpnI-PacI fragment and inserted it into the transfection plasmid, pR-CSPfFL-CD8CT. Lastly, XhoI and KasI were used to linearize pR-CSPfFL-CD8CT prior to transfection of GFP-Luciferase *P. berghei* ANKA parasites,^38^ as previously described ^39^ (Supplementary Fig. S4). In summary, the CSP amino acid sequence in the transgenic line consists of the signal sequence of the *P. berghei* CSP (amino acids 1 to 23) followed by residues 25 to 397 from the *P. falciparum* 3D7 CSP.

P.b.-P.f. CSP-FL CD8CT transgenic parasites were selected in Swiss Webster mice by treatment with pyrimethamine (MP Biomedicals, Solon, OH) in drinking water (0.07 mg/ml). Drug-resistant parasites were cloned by limiting-dilution. The successful recombination at the 5′ and 3′ ends of the modified locus was verified by PCR. The primers used to confirm 5′ integration were PbCS5′-F (TGCCCTATTCTCATATTTACCAC) and hDHRF5′ UTR-R (CACCATTTTTGAAAAGATTAATTTGA); the primers to verify integration at the 3′ end were PfCSP-F (TGAATGGTCCCCATGTAGTG) and PbCS3′UTR-R (CGCCTTAATGTGCATTGCT) (Supplementary Fig. S4). Finally, DNA isolated from transgenic parasites was sequenced to verify the replaced gene.

### Sporozoite challenge

To quantitatively assess liver parasite burdens, mice were challenged intravenously (i.v.) with 2 × 10^3^ chimeric P.b.-P.f. CSP-FL CD8CT parasites. Forty-two hours after sporozoite injection, liver parasite loads were measured by RT-qPCR.^40^

To evaluate sterile protection, mice were challenged by mosquito bites using five infected *A. stephensi* mosquitoes. Starting on day 4 after challenge, daily blood smears were taken and observed under a microscope. Smears were fixed with methanol (for 30 s) before staining with 10% Giemsa stain solution (Sigma-Aldrich, St. Louis, MO) for 15 min.

All sporozoite challenge experiments were performed 2 weeks after the last immunization.

### Statistics

Data were plotted using GraphPad Prism 6.0 software. Data were analyzed for significance using Kruskal–Wallis test, with Mann–Whitney *U* test for post hoc analysis. Analysis of flow cytometry data was performed using FlowJo software (TreeStar). No animals or samples were excluded from statistical analyses.

## Electronic supplementary material

Supplmental material
