# Supplementary material for: Robust antibody and CD8+ T-cell responses induced by P. falciparum CSP adsorbed to cationic liposomal adjuvant CAF09 confer sterilizing immunity against experimental rodent malaria infection
Source: NPJ Vaccines. 2017 Apr 10;2:10. doi: 10.1038/s41541-017-0011-y (PMC5603302; doi:10.1038/s41541-017-0011-y)
Supplement: Supplementary file 1 — Supplmental material [file 41541_2017_11_MOESM1_ESM.pdf]

(<u>SUPPLEMENTARY DATA</u>)

Robust antibody and CD8$^+$ T-cell responses induced by *P. falciparum* CSP adsorbed to cationic liposomal adjuvant CAF09 confer sterilizing immunity against experimental rodent malaria infection

Diego A. Espinosa,[a*] Dennis Christensen,[b] Christian Muñoz,[a] Sanjay Singh,[c] Emily Locke,[d] Peter Andersen,[b] Fidel Zavala [a#]

Department of Molecular Microbiology and Immunology, Johns Hopkins Malaria Research Institute, Johns Hopkins Bloomberg School of Public Health, Johns Hopkins University, Baltimore, Maryland, USA[a]; Department of Infectious Disease Immunology, Statens Serum Institut, Copenhagen, Denmark[b]; Gennova Biopharmaceuticals Ltd., Pune, India[c]; PATH Malaria Vaccine Initiative, Washington DC, USA[d]

Running Head: Protective antibody and CD8$^+$ T cells against malaria

*Present address: Diego A. Espinosa, Division of Infectious Diseases and Vaccinology, School of Public Health, University of California, Berkeley, Berkeley, California, USA

#Address correspondence to Fidel Zavala, fzavala1@jhu.edu

Keywords: malaria, vaccine, adjuvant, CD8$^+$ T cells, antibodies, *P. falciparum*, circumsporozoite protein

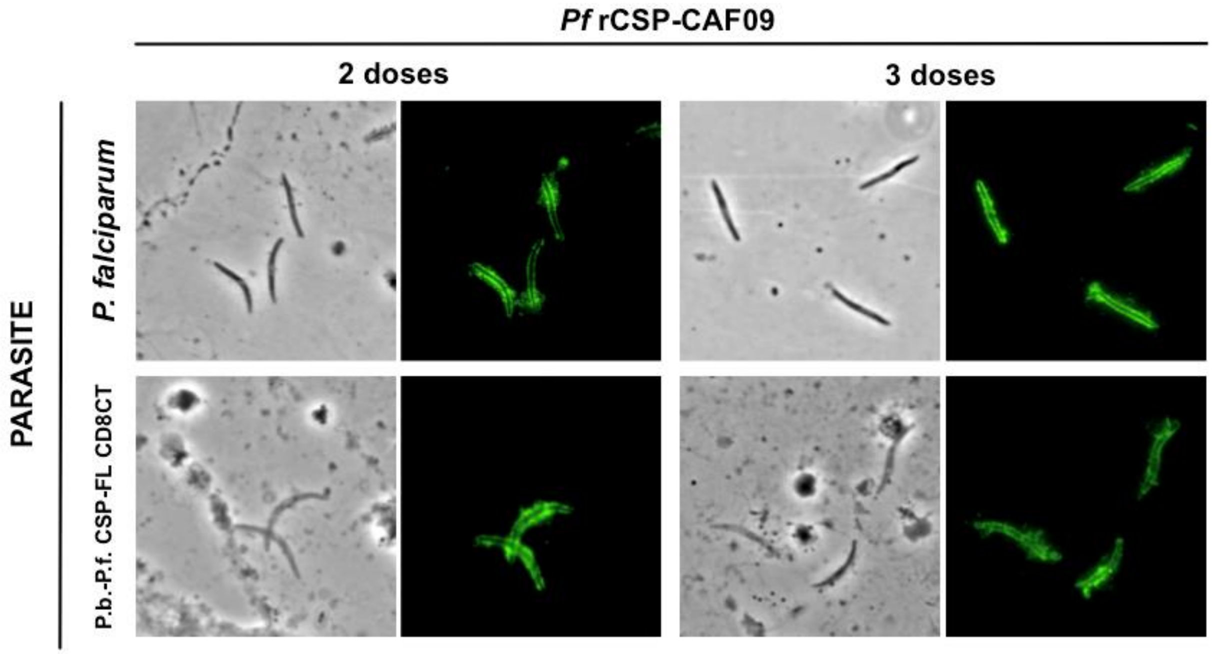

Supplementary figure 1 *Binding of polyclonal sera from Pf rCSP-CAF09-immunized mice to P. falciparum and P.b.-P.f. CSP-FL CD8CT transgenic sporozoites.* Sera from mice that received either 2 or 3 doses of Pf rCSP-CAF09 bind air-dried sporozoites with comparable efficiency, as determined by IFA. Images are representative of 1:16000 sera dilutions. Abbreviations: Pf rCSP-CAF09, *P. falciparum* recombinant circumsporozoite protein in adjuvant CAF09; P.b.-P.f. CSP-FL CD8CT, *P. berghei–P. falciparum* CSP full-length CD8 epitope C-terminus transgenic parasite; IFA, immunofluorescence assay.

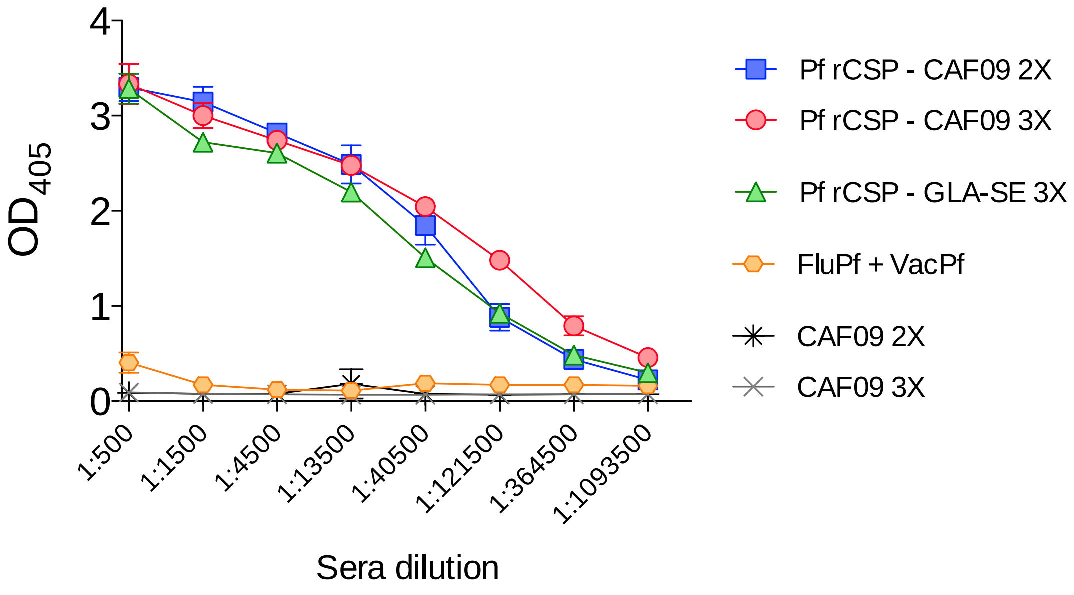

Supplementary figure 2 *Comparison of antibody titers against the Pf CSP repeat region.* Pooled serum samples from mice immunized with Pf rCSP-CAF09, Pf rCSP-GLA-SE, recombinant FluPf and VacPf viruses or CAF09 adjuvant (n=5 per group) were serially diluted and tested by ELISA against a synthetic peptide representing the *P. falciparum* CSP repeat region $[NANP]_7$. Anti-CSP IgG titers from mice immunized with Pf rCSP-CAF09 and Pf rCSP-GLA-SE were comparable, while FluPf and VacPf immunizations induced minimal antibody responses. Abbreviations: Pf rCSP-CAF09, *P. falciparum* recombinant circumsporozoite protein in adjuvant CAF09; Pf rCSP-GLA-SE, *P. falciparum* recombinant circumsporozoite protein in adjuvant GLA-SE; FluPf, recombinant Influenza virus expressing a region of the *P. falciparum* CSP C-terminus Pf; VacPf, recombinant Vaccinia virus expressing the full-length *P. falciparum* CSP; ELISA, enzyme-linked immunosorbent assay; OD, optical density.

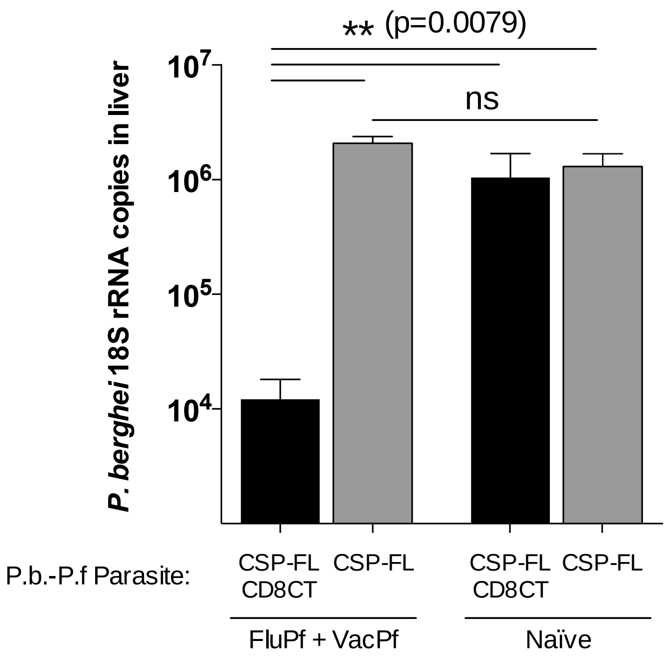

Supplementary figure 3 *Parasite liver burdens in mice immunized with recombinant FluPf and VacPf viruses.* Mice immunized with recombinant FluPf and VacPf viruses were challenged against transgenic *P. berghei-P. falciparum (P.b-P.f.)* parasites expressing the full-length *P. falciparum* 3D7 CSP containing the murine Class I MCH epitope $_{359}$DYENDIEKKI$_{368}$ (CSP-FL CD8CT) or the wild-type full-length *P. falciparum* 3D7 CSP (CSP-FL), which does not incorporate the cytotoxic epitope. Parasite liver loads of immunized mice challenged against P.b-P.f. CSP-FL CD8CT parasites were significantly lower than those of immunized mice challenged against P.b-P.f. CSP-FL sporozoites or naïve controls. Abbreviations: P.b.-P.f. CSP-FL CD8CT, *P. berghei–P. falciparum* CSP full-length CD8 epitope C-terminus transgenic parasite; P.f. CSP-FL, *P. berghei–P. falciparum* CSP full-length transgenic parasite; FluPf, recombinant influenza virus expressing *P. falciparum* CSP regions; VacPf, recombinant vaccinia virus expressing the *P. falciparum* CSP; rRNA, ribosomal RNA; RT-qPCR reverse-transcription quantitative real-time polymerase chain reaction.

A

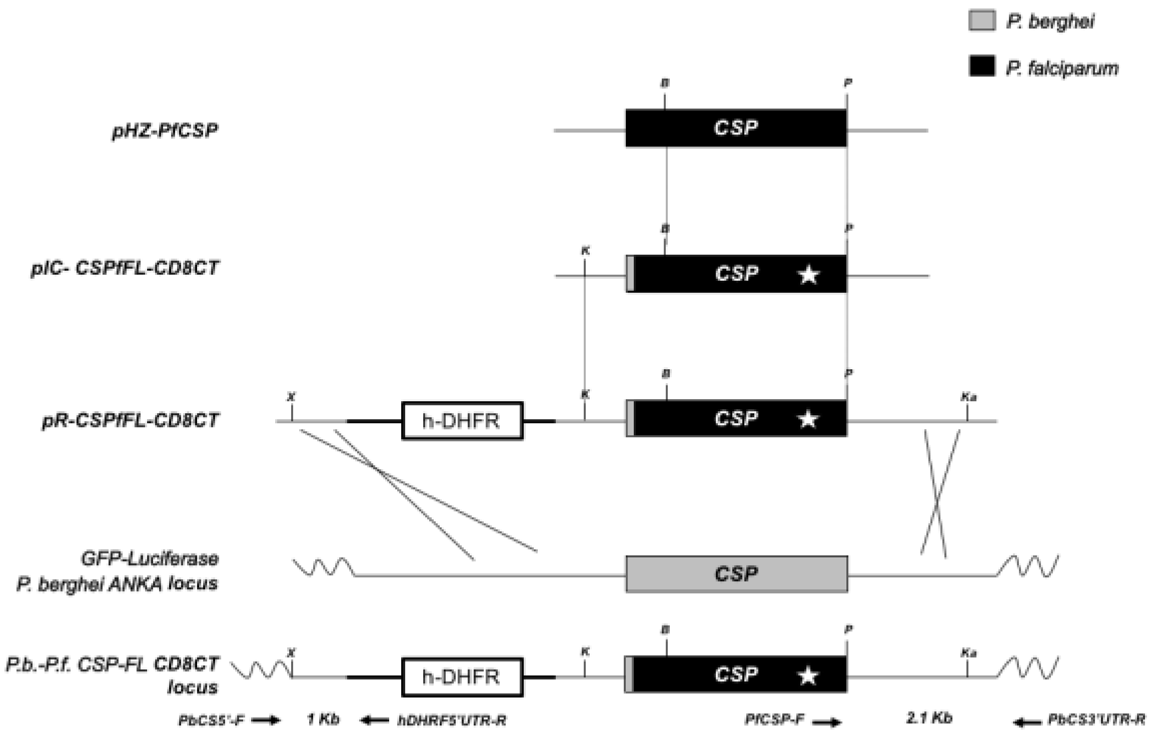

Supplementary figure 4 *Development of P.b.-P.f. CSP-FL CD8CT transgenic parasites.* (**A**) Scheme representing the strategy for replacing the CSP gene of GFP-Luciferase *P. berghei* (ANKA) with the *P. falciparum* (3D7) CSP incorporating the *P. berghei* signal sequence. The star symbol (☆) represents the location of the introduced nucleotide change to generate the $CD8^+$ T-cell epitope DYENDIEKKI. The annealing sites of the PCR primers used to verify recombination at the CSP locus are indicated below. Restriction sites shown are B – BbsI; K – KpnI; Ka – KasI; P – PacI; X – XhoI.

B

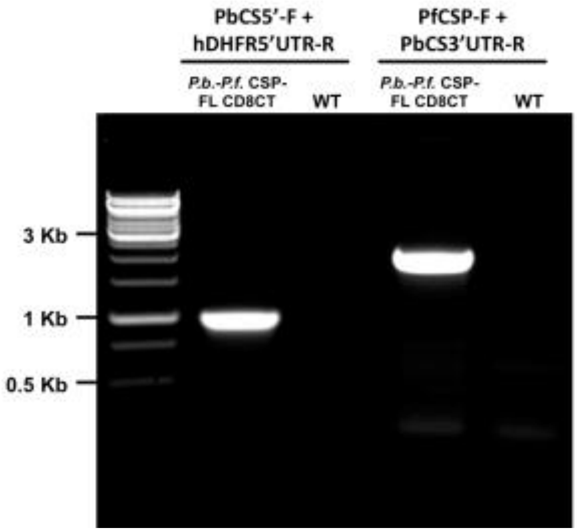

C

```
  1 MKKCTILVVA SLLLVNSLLP GYGCYGSSSN TRVLNELNYD NAGTNLYNEL 50
 51 EMNYYGKQEN WYSLKKNSRS LGENDDGNNE DNEKLRKPKH KKLKQPADGN 100
101 PDPNANPNVD PNANPNVDPN ANPNVDPNAN PNANPNANPN ANPNANPNAN 150
151 PNANPNANPN ANPNANPNAN PNANPNANPN ANPNANPNAN PNANPNVDPN 200
201 ANPNANPNAN PNANPNANPN ANPNANPNAN PNANPNANPN ANPNANPNAN 250
251 PNANPNANPN ANPNANPNAN PNKNNQGNGQ GHNMPNDPNR NVDENANANS 300
301 AVKNNNNEEP SDKHIKEYLN KIQNSLSTEW SPCSVTCGNG IQVRIKPGSA 350
351 NKPKDELDYE NDIEKKICKM EKCSSVFNVV NSSIGLIMVL SFLFLN*396
```

Supplementary figure 4 *Development of P.b.-P.f. CSP-FL CD8CT transgenic parasites.* (**B**) Using genomic DNA form P.b.-P.f. CSP-FL CD8CT transgenic parasites, 5’ and 3’ integration at the CSP locus was verified by PCR. Primers PbCS5’-F and hDHFR5’UTR-R yield a 1000 bp product; primers PfCSP-F and PbCS3’UTR-R yield a 2100 bp product. (**C**) Amino acid sequence of CSP in the P.b.-P.f. CSP-FL CD8CT parasite. The *P. berghei* signal sequence is depicted in gray text while the *P. falciparum* residues are represented in black text. The amino acid replacement (Ala (A) to Glu (E)) introduced to generate the cytotoxic epitope DYENDIEKKI is underlined.

Parameter	% Midgut infected	Oocysts/ midgut	% Salivary glands infected	Spz/Salivary gland (x 10^3)	% of infected mice after mosquito bites[B]
P.b.-P.f. CSP-FL CD8CT	**87.50**	**60.33**	**80**	**9.5**	**100%**
P. berghei ANKA	**80.20**	**55.25**	**75**	**10**	**100%**

Supplementary table 1 *Developmental characteristics and infectivity of P.b.-P.f. CSP-FL CD8CT transgenic parasites in A. stephensi mosquitoes*[A]. P.b.-P.f. CSP-FL CD8CT transgenic parasites have similar developmental characteristics as wild-type *P. berghei ANKA* parasites.

[A] Mean of 3 experiments, with at least 20 mosquitoes examined in each experiment.

[B] Three infected mosquitoes were allowed to feed on C57BL/6 mice (n=5) for 3 minutes. Blood stage parasitemia was assessed 5 days later.

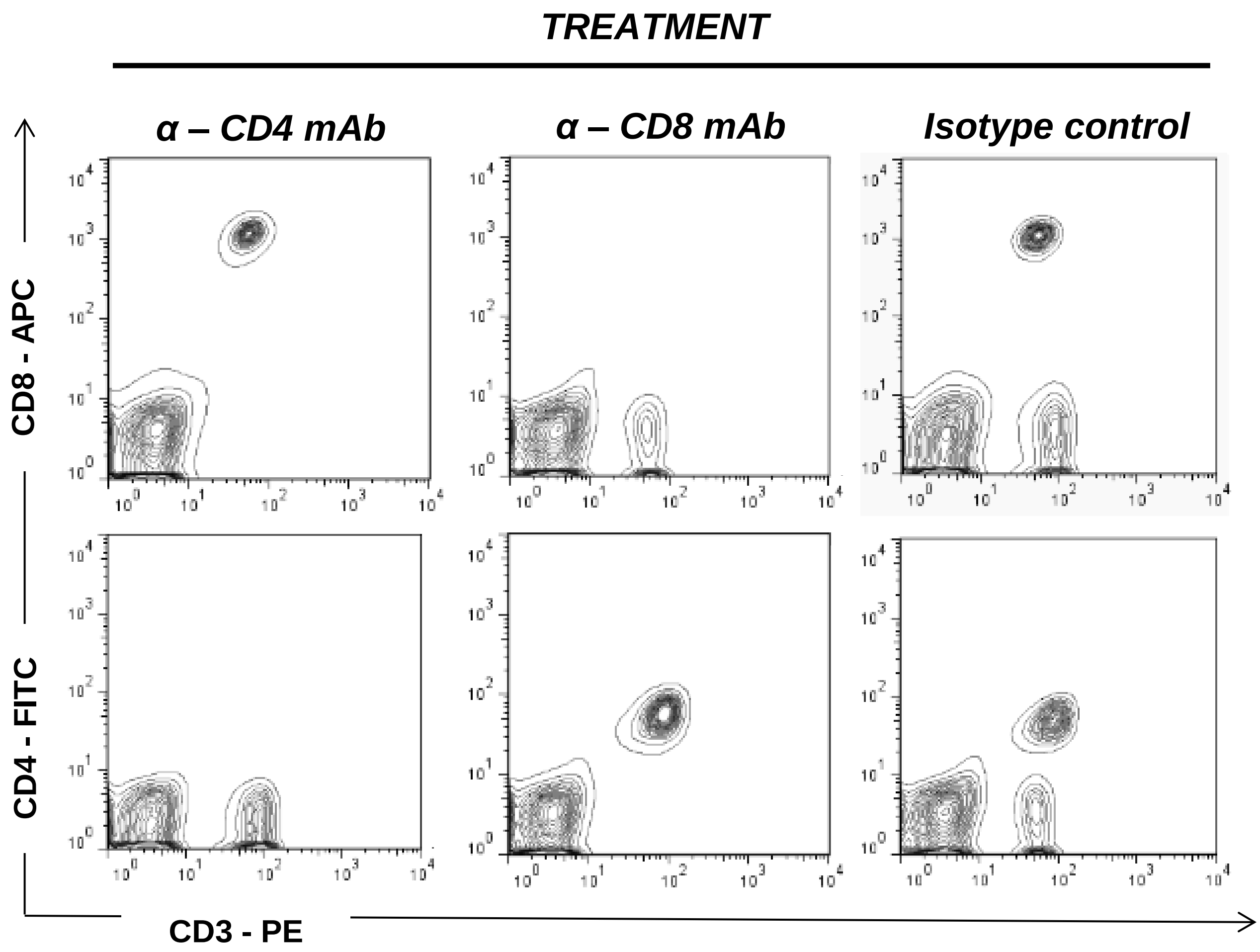

Supplementary figure 5 *Depletion of T cells by monoclonal antibody treatment.* Single-cell suspensions (5 x 10^5 cells) were harvested from the spleens of treated mice. Surface staining was performed with anti-CD3-PE, anti-CD4-FITC and anti-CD8-APC antibodies prior to flow cytometry analysis. Anti-CD4 and anti-CD8 antibody treatment efficiently depleted $CD4^+$ and $CD8^+$ T-cells, respectively. Abbreviations: α-CD4 mAb, anti-CD4 monoclonal antibody; α-CD8 mAb, anti-CD8 monoclonal antibody; PE, Phycoerythrin; FITC, Fluorescein isothiocyanate; APC, Allophycocyanin.
